# Development and Validation of LC-MS/MS Methods for Quantification of Fluralaner and Moxidectin in Cat Plasma and Its Application in a Pharmacokinetic Study

**DOI:** 10.3390/ani16091420

**Published:** 2026-05-06

**Authors:** Xiaolin Liu, Tianqi Huang, Xinggang Tang, Qianni Ye, Minggui Yuan, Zehua Cui, Yuqiao Ma, Junheng He, Rong Xiang

**Affiliations:** 1 Institute of Animal Health, Guangdong Academy of Agricultural Sciences, Guangdong Province Key Laboratory of Livestock Disease Prevention, Scientific Observation and Experiment Station of Veterinary Drugs and Diagnostic Techniques of Guangdong Province, Ministry of Agriculture and Rural Affairs, Guangzhou 510640, China; hndb2025001@126.com (X.L.); huangtianqi@gdaas.cn (T.H.); tangxinggang@gdaas.cn (X.T.); yeqianni@gdaas.cn (Q.Y.); yuanminggui@gdaas.cn (M.Y.); cuizehua@gdaas.cn (Z.C.); hjunhen@163.com (J.H.); 2 College of Veterinary Medicine, South China Agricultural University, Guangzhou 510642, China; mayuqiao@scau.edu.cn

**Keywords:** fluralaner, moxidectin, LC-MS/MS, cat plasma, pharmacokinetics

## Abstract

This study was conducted to support the development of a novel generic veterinary formulation by performing a comparative pharmacokinetic analysis against an approved reference product (Bravecto^®^ Plus). Sensitive and selective liquid chromatography-tandem mass spectrometry (LC-MS/MS) methods were developed and validated for the quantification of fluralaner and moxidectin in cat plasma. A single topical dose (40 mg/kg fluralaner and 2 mg/kg moxidectin) of either the test or reference formulation was administered to 20 healthy cats in a parallel study. A validated LC-MS/MS method was used to determine plasma concentrations. Key pharmacokinetic parameters—including peak plasma concentration (C_max_), time to C_max_ (t_max_), and area under the curve (AUC_0–∞_)—were calculated and statistically compared. The C_max_ of fluralaner was 1527.62 ± 905.94 ng/mL, t_max_ was 14.00 (interquartile range (IQR): 5.00–28.00) days and AUC_0–∞_ was 1,415,464.8 ± 822,873.6 ng·h/mL. The C_max_ of moxidectin was 27.85 ± 38.78 ng/mL, t_max_ was 6.00 (IQR: 1.00–14.00) days, AUC_0–∞_ was 16,807.2 ± 12,885.6 ng·h/mL. Compared with the reference veterinary drug (Bravecto^®^ Plus), the relative bioavailability (F) of both fluralaner (118.89% (90% CI: 96.5–145.3%)) and moxidectin (102.00% (90% CI: 85.2–118.8%)) exceeded 100%, and there was no statistical difference in the time to peak concentration (t_max_). The test and reference formulations exhibit similar pharmacokinetic profiles in cats. These results provide critical pharmacokinetic data supporting the bioequivalence and therapeutic potential of the new generic formulation, thereby facilitating its further development and regulatory evaluation.

## Simple Summary

Fluralaner and moxidectin are two drugs used together in a single spot-on product to protect cats from parasites such as fleas, ticks, and worms. This study develops and tests two sensitive methods to measure the levels of these drugs in cat blood. The methods are then used to compare a new generic product with an already approved reference product. The results show that the two products behave very similarly in the body, with no significant differences in how they are absorbed, distributed, or eliminated. The generic product released 118.89% of fluralaner and 102.00% of moxidectin compared to the reference product, confirming that the two formulations are bioequivalent. These findings support the use of the new generic product as an effective and equivalent alternative for parasite control in cats.

## 1. Introduction

Parasitic diseases represent a common clinical concern in dogs and cats. Various endo- and ectoparasites can adversely affect the host through multiple mechanisms, including deprivation of nutrients, damage to tissues and cells, secretion of toxins, and impairment of growth and development [1,2]. These effects pose a significant threat to the health of companion animals, even a potential risk to public health. Given the diversity of parasitic species, their prevention and control are considerable challenges. Infections caused by parasites can lead to clinical disease, with severe cases potentially resulting in mortality [3,4]. For the management of parasitic infections in pets, compound formulations are currently considered a therapeutic choice. Examples include combinations such as fluralaner and moxidectin, milbemycin oxime and praziquantel, and imidacloprid and moxidectin, facilitating the simultaneous management of infestations and improving therapeutic outcomes [5,6].

Fluralaner is a systemic ectoparasiticide with the selective inhibition of ligand-gated chloride channels, specifically γ-aminobutyric acid (GABA) and glutamate, in the peripheral and central nervous systems of parasites [7,8]. Toxicology studies confirm the safety profile of fluralaner, showing no adverse effects at doses well above the therapeutic range [9]. A single oral dose of fluralaner is quickly absorbed and persists in the body for an extended period due to its wide distribution and slow clearance [10]. The long-lasting activity of a single fluralaner dose against fleas and ticks in dogs and cats is a direct result of these pharmacokinetic properties [11]. Moxidectin is a second-generation macrocyclic lactone derived from nemadectin, with broad-spectrum activity against nematodes and arthropods. It is used in veterinary medicine to treat and prevent intestinal worms and heartworm in a variety of animals [12]. The pharmacokinetic profile of moxidectin is characterized by high distribution into lipid tissues, a large volume of distribution, and low clearance, which result in a long terminal half-life [13]. These features allow moxidectin to be suitable for long-acting formulations and to be a better control of parasites [14].

A combined spot-on formulation of fluralaner and moxidectin has received US FDA approval in 2019 for use in cats for the prevention of heartworm disease (Dirofilaria immitis) and the treatment of intestinal nematode infections, specifically roundworms and hookworms [15]. BRAVECTO^®^ PLUS inhibits adult fleas and was applied for the treatment and prevention of flea infestations (Ctenocephalides felis) and tick infestations (Ixodes scapularis (black-legged tick) and Dermacentor variabilis (American dog tick)) for 2-month-old kittens and with 6-month or older cats [16,17,18]. This established efficacy profile makes it the pivotal reference product for the development of bioequivalent generic alternatives.

To support the development of a new generic veterinary drug, this study addresses a key analytical and pharmacokinetic challenge: the simultaneous quantification of fluralaner and moxidectin. We conducted a comparative pharmacokinetic analysis in cats following a single spot-on application of either the investigational drug (Guangdong Kangyu Biotechnology, Guangzhou, China) or the reference drug (Bravecto Plus^®^, MSD, Rahway, NJ, USA). The study was designed to develop and validate a sensitive LC-MS/MS method for simultaneous quantification of fluralaner and moxidectin in cat plasma. We applied this method in a comparative pharmacokinetic study to support generic drug development.

## 2. Method

### 2.1. Animals

Twenty healthy British Shorthair cats (aged 1.3–4.9 years, with a body weight of 3.17 ± 0.25 kg, and an equal gender distribution) were selected for this study. As this was an exploratory pharmacokinetic study without prior internal pilot data, the sample size was determined based on established practices in veterinary PK research and literature-derived estimates [10,11]. The experimental procedures were conducted at the Experimental Animal Center of South China Agricultural University (Approval license number: 2024e003). Before the start of the test, a 7-day adaptation period at the center was performed for the test animals and a physical examination was performed by the veterinary staff. The environmental conditions of the cattery house are as follows: temperature: 20~25 °C, relative humidity: 65%~75%, light/dark time: 12 h/12 h. All test animals were given quantitative commercial pellet feed (Ramical Animal Nutrition and Healthcare Technology Co., Ltd., Foshan, China) daily with a nutritional composition value: Crude Protein 28%, calcium 1%, crude fiber 5%, water 10%, crude fat 11%, crude ash 10%, phosphorus 0.8%, methionine 0.29%. The feed was provided twice daily, in the morning and evening, with ad libitum access to water through the drinking water tank placed in each cat cage. The quality of drinking water is in line with the relevant national regulations on human water use, etc.

### 2.2. Design, Drug Administration and Sampling

The study was administered in a randomized (computer-generated random number list) single dose, with a parallel trial design for both formulations’ treatments. The drug was administered at a single, topical skin dose of 40 mg/kg (fluralaner) and 2 mg/kg (moxidectin). The test cats fasted overnight after dinner the day before the administration. The dosage of the test preparation was calculated according to the weight weighing results of the day before the administration; all animals were fed a standardized diet commencing 4 h after the drug administration. Random numbers were generated using the EXCEL (v 16.0) random number generator and sorted in ascending order, and 20 cats were randomly divided into two groups: the subject drug group and the control group.

Cats were administered in single doses according to the trial design. The blank samples were collected before administration, and after topical administration, 24 blood samples for each test cat were collected at 0 h, 8 h, 12 h, 1 d, 2 d, 3 d, 5 d, 7 d, 10 d, 14 d, 21 d, 28 d, 35 d, 42 d,49 d, 56 d, 63 d, 70 d, 77 d, 84 d, 98 d, 105 d and 112 d. At each time point, 4 mL of blood from the cephalic vein of the forelimb was collected. All the collected blood samples were temporarily stored in a 4 °C refrigerator and upper plasma samples were obtained by centrifugation at 4000 rpm for 10 min within 2 h after collection. The plasma layers were collected as the samples to be tested.

### 2.3. Sample Preparation

After collection, the samples were centrifuged at 4000 rpm for 10 min at room temperature to separate the plasma. The plasma was divided into test samples and reserve samples, which were stored in a −40 °C freezer in the sample chamber.

For analysis, the frozen samples were thawed naturally at room temperature. A precise volume of 200 μL plasma was aliquoted, and 20 μL of a mixed internal standard working solution (containing fluralaner-D4 at 5000 ng/mL and moxidectin-D3 at 500 ng/mL) was added and vortexed briefly. Then, 780 μL of acetonitrile was added and vortexed for 1 min. The samples were centrifuged at 14,000 rpm for 10 min at 4 °C. The 800 μL supernatant was carefully collected and processed in two parallel streams: The supernatant intended for moxidectin analysis was passed through an E-Gen phospholipid removal 96-well plate. The supernatant intended for fluralaner analysis was processed without this phospholipid removal step. Both supernatants were then individually filtered through 0.22 μm polytetrafluoroethylene pink filters. The filtrates were collected into 200 μL internal vials (inserts) and analyzed using two independent liquid chromatography-tandem mass spectrometry (LC-MS/MS) systems.

### 2.4. Instrument and Conditions

The fluralaner analysis was performed using an Athena UPLC C18 column (2.1 mm × 50 mm, 1.8 μm). A gradient elution was applied at a flow rate of 0.4 mL·min^−1^ with a mobile phase consisting of 0.1% formic acid in water and acetonitrile. The gradient program was as follows: 0–2.10 min, 20% B to 99% B; 2.10–5.50 min, 99% B; 5.50–5.60 min, 99% B to 20% B; 5.60–8.50 min, 20% B. The column temperature was maintained at 40 °C, and the injection volume was 5 μL.

Mass spectrometric detection was conducted using an Agilent 1200 liquid chromatography system coupled with an API 4000 mass spectrometer (Framingham, MA, USA) operated in multiple reaction monitoring (MRM) mode. An electrospray ionization (ESI) source was used in positive ion mode. The following transitions were selectively monitored: *m*/*z* 556.3 > 400.1, *m*/*z* 556.3 > 457.0 for fluralaner, and *m*/*z* 560.0 > 402.2 for the internal standard (deuterated analog). The concentration of fluralaner in cat plasma was quantified using the internal standard method.

The moxidectin analysis was performed using an Athena UHPLC C18 column (2.1 mm × 50 mm, 1.8 µm) with a mobile phase consisting of 0.1% formic acid in water and acetonitrile. Gradient elution was carried out at a flow rate of 0.4 mL/min under the following program: 0–1.00 min, 20% B; 1.00–1.50 min, 20% B → 85% B; 1.50–3.50 min, 85% B → 95% B; 3.50–5.50 min, 95% B; 5.50–5.60 min, 95% B → 20% B; 5.60–8.50 min, 20% B. The column temperature was maintained at 40 °C, and the injection volume was 5 µL. Mass spectrometric detection was conducted using a Multiple Reaction Monitoring (MRM) scan mode with an electrospray ionization (ESI) source (Shimadzu Scientific Instruments, Inc., Tokyo, Japan) in positive ion mode. The selective monitoring was set for the following mass-to-charge ratios (*m*/*z*): 640.40/528.15*, 640.40/498.40 for moxidectin, and 643.50/531.30 for its deuterated internal standard. LC-MS/MS analysis was performed using a Shimadzu LCMS-8050 ultra-high performance liquid chromatography triple quadrupole mass spectrometer. The concentration of moxidectin in cat plasma was quantified using the internal standard method.

### 2.5. Preparation of Standards and Quality Control Samples

Moxidectin standard (purity: 99.2%, batch number: 7283M, certificate available) was purchased from China National Standard Pharmaceutical Co., Ltd., Beijing, China. Fluralaner standard (purity: 99.5%, batch number: 2,426,646, certificate available), Fluralaner-d4 (purity: 98.8%, batch number: 2,414,338), and Moxidectin-d3 (purity: 99.0%, batch number: 2,432,061) were purchased from Anpel, Shanghai, China. Certificates of analysis were provided by the manufacturer. Methanol, acetonitrile, and formic acid (HPLC grade) were obtained from Anpel, Shanghai, China). Deionized water was purified using a water purification system.

Stock solutions of fluralaner, moxidectin, fluralaner-D4 (IS), and moxidectin-D3(IS) were prepared in methanol. On the day of analysis, working solutions were added to blank plasma to prepare calibration standards and quality control samples. Matrix-matched calibration standards were prepared by spiking blank cat plasma with eight non-zero standards concentrations of fluralaner from 5 ng/mL to 2500 ng/m and moxidectin from 0.5 ng/mL to 250 ng/mL. All spiked samples were processed using the same extraction procedure as the study samples. Quality control samples were prepared by spiking blank cat plasma with known concentrations of fluralaner and moxidectin at 4 levels (5, 15, 800 and 2000 ng/mL for fluralaner; 0.5, 1.5, 80 and 200 ng/mL for moxidectin) to evaluate the accuracy, precision, and stability of the method validation. Working standard solutions were stored at 4 °C. Inventories and working solutions for both calibration standards and spiked plasma samples were separately prepared.

### 2.6. Method Validation

#### 2.6.1. Limit of Detection (LOD)

Blank plasma aliquots (2 mL centrifuge tubes) were spiked with an appropriate volume of internal standard (IS) working solution and used immediately. The plasma samples with different spiking nominal fluralaner concentrations (2.5 ng/mL and 5 ng/mL) and nominal moxidectin concentrations (0.25 ng/mL and 0.5 ng/mL) were prepared for detection. Based on the minimum detectable concentration, the spiked sample concentration with a signal-to-noise ratio S/N ≥ 3 is defined as the limit of detection (LOD), while the concentration with S/N ≥ 10 is defined as the limit of quantification (LOQ).

#### 2.6.2. Selectivity

Blank plasma samples from six different cats were individually injected and analyzed to assess interference from endogenous substances. Six individual blank plasma samples with working solution and IS were prepared at the lower limit of quantification (LLOQ). The peak area of any co-eluting endogenous signal (interference peak) within ±0.2 min of the analyte’s retention time was detected. At the retention times of the target analyte, the response of endogenous substances (disruption peak) in all blank plasma samples was less than 20% of the response at LLOQ and lower than 5% of IS. These acceptance criteria in method validation are under the guidance of Veterinary Pharmacopeia of the People’s Republic of China (Administration, 2020).

#### 2.6.3. Carry-Over

Blank plasma with the corresponding concentration of fluralaner and moxidectin working solution was prepared for immediate use, to obtain an upper limit of quantification (ULOQ), matrix-spiked sample of fluralaner (1500 ng/mL) and moxidectin (150 ng/mL). Carry-over was evaluated by sequentially injecting an ULOQ sample, a blank plasma sample, and another LLOQ plasma sample with IS. After each analysis of a high-concentration spiked sample, a blank mobile phase sample was injected to monitor and prevent carry-over. Six replicates were performed to assess the carry-over in the detection system. The response of the analyte in the blank plasma following the ULOQ sample was less than 20% of the LLOQ response and 5% of the response of IS, which can detect whether significant carry-over took place.

#### 2.6.4. Matrix Effect

Six individual blank plasma samples with working solution and IS were prepared for fluralaner (15 and 2000 ng/mL) and moxidectin (1.5 and 200 ng/mL) as matrix samples. Six high-dose samples and low-dose samples in pure solution with working solution and IS were prepared for fluralaner (15 and 2000 ng/mL) and moxidectin (1.5 and 200 ng/mL). Matrix effects were assessed by comparing the response of matrix samples with those of pure solutions at identical concentrations and by a normalized matrix factor compared with IS. The normalized matrix factor precision (coefficient of variation, CV) should generally be ≤15%.

#### 2.6.5. Linearity

Calibration standards were prepared fresh on each of the three validation days by spiking blank cat plasma with appropriate working solutions to generate matrix-matched calibration curves containing both analytes. The final nominal concentrations were 5, 10, 50, 100, 500, 1000, 1500, 2000, and 2500 ng/mL for fluralaner and 0.5, 1, 5, 10, 50, 100, 150, 200, and 250 ng/mL for moxidectin. All calibration standards were processed immediately following the same extraction procedure described for study samples, with each concentration prepared and analyzed in triplicate per validation run. The remaining aliquot (0.3 mL) served as the blank control. A calibration curve was constructed by plotting the chromatographic peak area of samples (*Y*-axis) against the corresponding nominal concentration (*X*-axis), from which the regression equation and correlation coefficient (r^2^) were derived. The standard curve correlation coefficient (r^2^) should generally be ≥0.99, which indicates the standard curve is within requirement.

#### 2.6.6. Accuracy and Precision

Blank plasma with certain working solution was prepared for immediate use at four final concentration levels of fluralaner (5, 15, 800 and 2000 ng/mL) and moxidectin (0.5, 1.5, 80 and 200 ng/mL). Six replicates were performed for each concentration. The samples were processed and analyzed under the instrumental conditions specified in Section 2.4. The experiment was conducted over three separate working days, with one batch prepared and analyzed each day. Quantification was performed using a matrix-matched standard curve prepared immediately. Intra-day (within-batch) and inter-day (between-batch) accuracy and precision were calculated. The mean accuracy should be within ±15% of the nominal value of the quality control samples, and the accuracy at the lower limit of quantification (LLOQ) should be within ±20% of the nominal value. The intra-day precision (CV) should generally be ≤15%, and the CV at the LLOQ should be ≤20%.

#### 2.6.7. Recovery

Blank plasma with working solution was prepared for immediate use at three nominal concentrations of fluralaner (15, 800 and 2000 ng/mL) and moxidectin (1.5, 80 and 200 ng/mL) and then the spiked plasma samples were processed. Another 0.3 mL of processed blank plasma with 0.6 mL of working solution was prepared for immediate use before use at three nominal concentrations of fluralaner (15, 800 and 2000 ng/mL) and moxidectin (1.5, 80 and 200 ng/mL). Six replicates for each concentration were detected and analyzed. The peak area values for spiked samples and processed samples were examined. Using the processed samples as the reference, the recovery rates of the spiked samples for fluralaner and moxidectin were calculated. The recovery ratio should be more than 70% and the precision (CV) should be ≤15%.

#### 2.6.8. Stability

To evaluate stability, samples of fluralaner (nominal concentrations of 15 and 2000 ng/mL) and moxidectin (nominal concentrations of 1.5 and 200 ng/mL) were prepared. The stability in process was analyzed, including stability in whole blood sample (2 h at room temperature) and in plasma for 24 h at room temperature. The stability in biological matrix was analyzed, including stability for long-term storage (−40 °C) for 1, 3 and 7 months and stability for three freeze–thaw cycles (24 h per cycle from −20 °C to room temperature). The stability in processed plasma was analyzed for 24 h at refrigerator (4 °C), sample tray (8 °C) and room temperature (25 °C) conditions. Six replicates per concentration were subjected to all 9 tests. Subsequently, test fluralaner and moxidectin concentrations were measured and compared with freshly prepared samples via a fresh-prepared standard curve. The stability precision (CV) should generally be ≤15%, which indicates its stability under these storage or processing conditions.

#### 2.6.9. Dilution Integrity

To evaluate dilution integrity, blank plasma with working solution was prepared for immediate use at nominal fluralaner concentrations (4000 ng/mL), and samples with 10-fold dilution concentrations were prepared by diluting with pre-processed blank plasma, with six replicates for each concentration level. The dilution integrity precision (CV) should generally be ≤15%, which indicates its stability under the dilution conditions.

### 2.7. Pharmacokinetics

All cats were handled with due regard for their welfare and in compliance with all local and national regulatory requirements. The pharmacokinetic profiles of fluralaner and moxidectin were studied in 20 cats aged ≥12 months, weighing between 2.8 and 3.6 kg. Prior to PK studies, these cats were fasted overnight and were allowed free access to water. Cats were treated at the minimum recommended dose rate of 40 mg fluralaner plus 2 mg moxidectin/kg based on body weight. A single dose of 40 mg/kg fluralaner and 2 mg/kg moxidectin formulation was administered in one or several spots dorsally along the cat’s back. Blood samples of 2 mL were drawn from the antecubital vein and collected in tubes containing heparin at designated time points. The blood samples were centrifuged at 4000 rpm for 10 min at 2–8 °C to separate plasma, then stored at −80 °C until analysis. Plasma PK parameters of fluralaner and moxidectin were measured by non-compartmentalized analysis in Phoenix WinNonlin^®^ 8.4 (Certara, L.P., Princeton, NJ, USA). While 20 cats were enrolled, samples from specific time points were unavailable due to an insufficient AUC value. Consequently, 1 individual (in Group A) in the PK table of fluralaner and 3 individuals (2 in Group A and 1 cat in Group B) in the PK table of moxidectin were excluded with analyzable data for each respective time point or parameter. During the pharmacokinetic analysis, data from certain animals were excluded based on pre-defined criteria established. Animals that missed more than two consecutive scheduled blood collection time points due to technical issues (catheter failure, sample hemolysis) were excluded from the final PK analysis (*n* = 1 from the reference group).

### 2.8. Statistical Analysis

All data was expressed as mean ± SD of each independent replication. Pharmacokinetic parameters were determined via a non-compartmental model (NCA) with Phoenix WinNonlin^®^ 8.4. Individual and mean plasma concentration time curves were plotted for each animal. Concurrently, the relative bioavailability of the test veterinary drug group was calculated based on the AUC_0–t_ results of the control veterinary drug group using Excel. The calculation employed the following formula:

F = (AUC_T_ × D_R_/AUC_R_ × D_T_) × 100%

The AUC_0–∞_ (area under the curve from time zero to infinity) was used to calculate relative bioavailability (F). AUC_T_ and AUC_R_ represent the area under the curve for the test (T) and reference (R) formulations, respectively. D_T_ and D_R_ represent the doses administered for the test (T) and reference (R) formulations, respectively.

The main pharmacokinetic parameters (C_max_, AUC_0–t_, AUC_0–∞_) of the test drug and the control drug group were logarithmically transformed, along with the remaining pharmacokinetic parameters, and then analyzed via WinNonlin^®^ 8.4. For continuous data that followed a normal distribution (*p* > 0.05) and exhibited homogeneity of variance (*p* > 0.05), an independent samples *t*-test (a parametric test for two independent groups) was applied. For continuous data that did not follow a normal distribution (*p* < 0.05), regardless of the variance homogeneity result (*p* > 0.05 for homogeneity or *p* < 0.05 for heterogeneity), the Mann–Whitney U test (a non-parametric rank-sum test for two independent groups) was employed. When *p* > 0.05 in the 95% CI, no significant differences between two groups were observed.

## 3. Results

### 3.1. Method Validation

#### 3.1.1. Limit of Detection (LOD)

The signal-to-noise ratio (S/N) of fluralaner samples at the LOD concentration level ranged from 161.35 to 275.39, while at the LOQ concentration level, it ranged from 280.68 to 749.80 with the average accuracy of 7.84% and CV% of 11.87% (Table 1). The signal-to-noise ratio (S/N) of moxidectin samples at the LOD concentration level ranged from 3.76 to 10.97, while at the LOQ concentration level, it ranged from 11.74 to 42.38 with the average accuracy of 2.00% and CV% of 3.92%. The results show that the signal-to-noise ratios for both the LOD and LOQ met the criteria of ≥3 and ≥10, respectively. Furthermore, the accuracy of the LOQ was within ±20% of the nominal value, and precision (CV%) was ≤20%. These findings indicate that the LOD and LOQ of the method meet the requirements for bioanalytical applications.

#### 3.1.2. Selectivity

**Figure 1:**
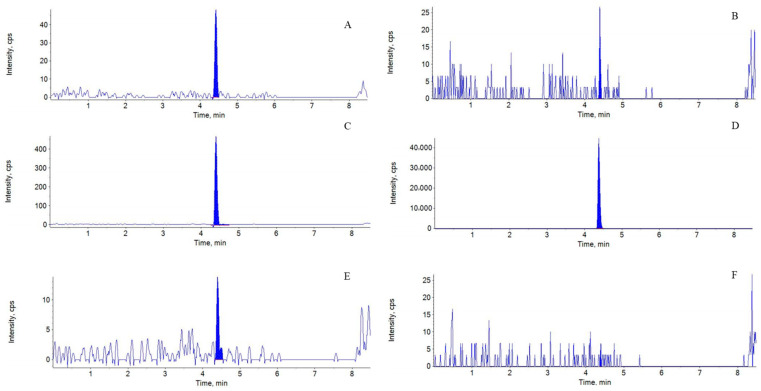
Representative MRM chromatograms of fluralaner in blank plasma (**A**,**B**), blank plasma spiked with LLOQ (**C**), blank plasma spiked with IS (**D**), carry-over effect of fluralaner (**E**) and carry-over effect of IS (**F**).

**Figure 2:**
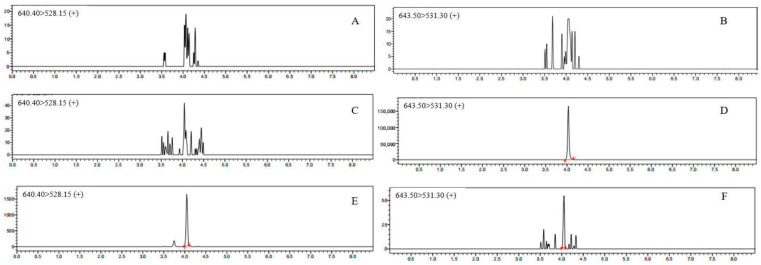
Representative MRM chromatograms of moxidectin in blank plasma (**A**,**B**), blank plasma spiked with LLOQ (**C**), blank plasma spiked with IS (**D**), carry-over effect of moxidectin (**E**) and carry-over effect of IS (**F**).

The representative chromatograms of blank plasma, blank plasma spiked with LLOQ and the internal standard are shown in Figure 1 (fluralaner) and Figure 2 (moxidectin). The ratio of the average disruption peak of the analyte at the retention time ranged from 7.21% to 10.97% (Table 2; Figure 1A,C). The ratio of the average disruption peak of the LLOQ label at the IS retention time ranged from 0.01% to 0.03% (Table 2; Figure 1B,D). The average interference peak ratio to the analyte at the retention time of moxidectin was between 3.68% and 8.13% (Table 3; Figure 2A,C). The ratio of the peak area of the interference peak at the retention time of the internal standard to the peak area of the LLOQ sample internal standard was between 0.03% and 0.05% (Table 3; Figure 2B,D). The absence of significant chromatographic interference from the plasma matrix at the retention times corresponding to fluralaner, moxidectin, and the internal standard (IS) validates the selectivity of the developed MRM method.

#### 3.1.3. Carry-Over

The carry-over effect was evaluated by sequentially injecting a blank plasma sample immediately following the analysis of a sample at the upper limit of quantification (ULOQ). No carry-over effects at the retention time of fluralaner were detected when blank plasma samples were analyzed (Table 4; Figure 1C,E). Intensities of blank plasma samples of 0.81%~5.48% for blank and within 0.01% for IS were below the required limits of 20% and 5% of the LLOQ samples (Table 4; Figure 1D,F).

No analyte was detected at the retention time of moxidectin in the carry-over blank sample (Table 4; Figure 2C,E). The peak area of the internal standard in the carry-over blank sample at the internal standard’s retention time was within 0.03% of the internal standard peak area in the LLOQ sample (Table 4; Figure 2D,F).

#### 3.1.4. Matrix Effect

The matrix effects for fluralaner and moxidectin in cat plasma were quantitatively assessed following normalization with an internal standard (Table 5). The average calculated normalized matrix effect factors for fluralaner were 1.025 with CV (%) of 4.78% at the concentration of 15 ng/mL and 1.024 with CV (%) of 3.91% at the concentration of 2000 ng/mL. In addition, the average calculated normalized matrix effect value for moxidectin was 0.856 with CV (%) of 4.67% at the concentration of 1.5 ng/mL and 0.944 with CV (%) of 2.86% at the concentration of 200 ng/mL. These data indicate that no significant matrix effect was observed under the experimental conditions.

#### 3.1.5. Linearity

The standard curve was calculated using a weighted method to obtain the respective regression equations and correlation coefficients (*R*^2^). The standard curves exhibited good linearity within the range of 5–2500 ng/mL, with *R*^2^ values ranging from 0.9943 to 0.9998, averaging 0.9975 (Table 6). The standard curves exhibited good linearity within the range of 0.5–250 ng/mL, with *R*^2^ values ranging from 0.9967 to 0.9983, averaging 0.9975. These data indicate that a standard curve meets the requirements under the experimental conditions.

#### 3.1.6. Accuracy and Precision

Intra-day (Table 7) and inter-day (Table 8) precision and accuracy of fluralaner and moxidectin were analyzed. Intra-day precisions of fluralaner ranged from 1.90% to 11.31% with accuracy levels of 86.31% to 110.20% and intra-day precisions of moxidectin ranged from 1.57% to 9.15% with accuracy levels of 91.33% to 107.63%, respectively. Inter-day precisions of fluralaner were from 3.49% to 11.63% with accuracies of 89.77–103.61% and the inter-day precisions of moxidectin were from 6.16% to 7.62% with accuracies of 96.00–101.16%, respectively. The intra-day precision (CV) general was ≤15%, and the CV at the LLOQ was ≤20%. These data indicate that accuracy and precision meet the requirements under the experimental conditions.

#### 3.1.7. Recovery

Table 9 shows the evaluation results of recoveries. After pretreatment by this method, the mean extraction recovery of fluralaner in cat plasma at three concentrations (15, 800, and 2000 ng/mL) was 96.05–99.94%. The mean extraction recoveries of moxidectin at 1.5, 80, and 200 µg/mL were 103.06–107.73%. The average extraction yields of fluralaner-D4 (10,000 ng/mL) and moxidectin-D3 (1000 ng/mL) were 96.23% and 74.11%, respectively. The CV (%) value of the estimated extraction rate was within ±15%, indicating a high reproducibility of the sample preparation process.

#### 3.1.8. Stability

Stability tests (Table 10) show that fluralaner and moxidectin were stable in the whole blood sample (2 h at room temperature) and in plasma for 24 h at room temperature. The biological matrix indicates stability as well, including stability for long-term storage (−40 °C) for 1, 3 and 7 months and stability for three freeze–thaw cycles. The processed sample was analyzed for stability for 24 h at refrigerator (4 °C), sample tray (8 °C) and room temperature (25 °C) conditions. Across all nine stability tests, the accuracy of all analytes ranged from 90.11% to 110.59%, with relative standard deviations ≤15%. An accuracy within ±15% across all tested levels is considered sufficient for storage stability of all analytes.

#### 3.1.9. Dilution Integrity

When plasma sample concentrations exceed the ULOQ, a 10-fold dilution was performed to quantify fluralaner in the cat plasma samples within the range of the standard curve (Table 11). The measured concentration of the samples was compared with the diluted nominal concentration (400 ng/mL), yielding an average accuracy deviation (%DEV) of 107.75% and a precision (coefficient of variation, %CV) of 3.10%. An accuracy within ±15% of the diluted fluralaner is considered sufficient for dilution integrity.

### 3.2. Application in a Pharmacokinetic Study in Cats

**Figure 3:**
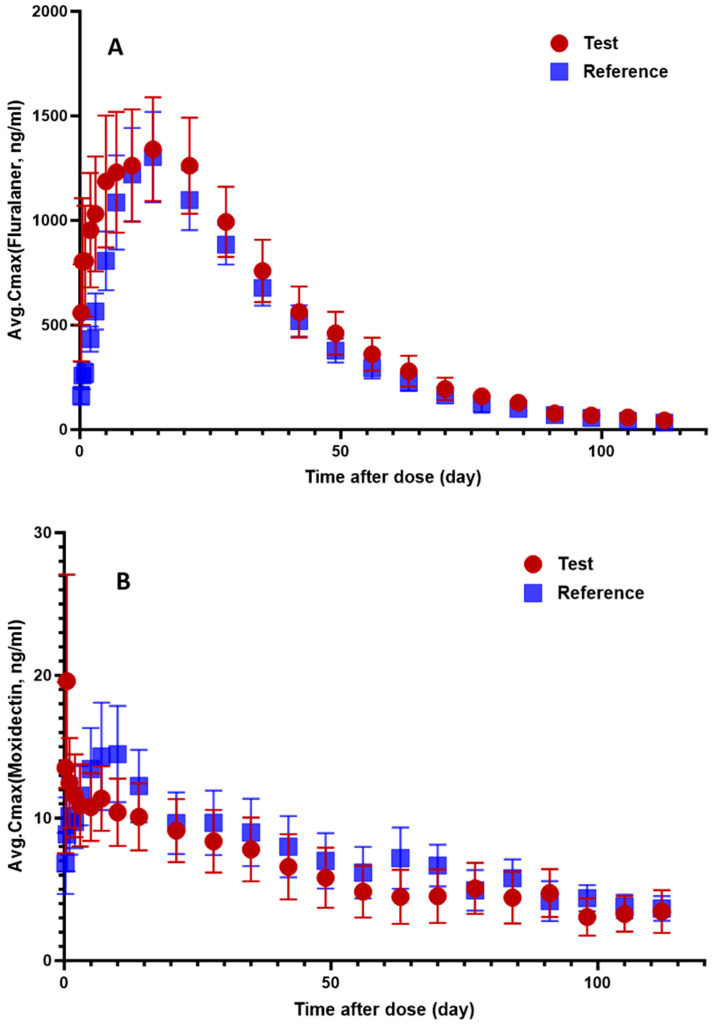
Plasma concentration–time profile of fluralaner (**A**) and moxidectin (**B**) in cats following administration of 40 mg fluralaner and 2 mg moxidectin. Data are presented as mean ± SD (standard deviation). The number of animals per group was *n* = 9–10 for each analyte. Circles represent the test group, and squares represent the reference group. Error bars indicate the standard deviation at each time point.

The mean plasma concentration–time profiles of fluralaner and moxidectin were characterized after cutaneous administration of 40 mg/kg fluralaner and 2 mg/kg moxidectin to cats (*n* = 20, Figure 3).

In the test fluralaner group, the pharmacokinetic parameters were comparable to those of the reference group. Half-life (t_1_/_2_) was 18.76 ± 6.85 d for the test group versus 17.23 ± 5.63 d for the reference group. Peak concentration (C_max_) values were 1527.62 ± 905.94 ng/mL and 1359.83 ± 675.63 ng/mL, respectively, with median tmax of 14.00 days for both groups. The area under the curve (AUC_0–∞_) was 1,415,464.8 ± 822,873.6 ng·h/mL for the test group and 1,190,565.6 ± 483,763.2 ng·h/mL for the reference group. The relative bioavailability (F) of the test formulation was 118.89%. All other parameters, including volume of distribution (Vd/F), systemic clearance (CL/F), and mean residence time (MRT), showed similar values between groups, as detailed in Table 12.

In the test veterinary drug group of moxidectin (Table 13): The half-life (t_1/2_) of moxidectin was 18.77 ± 12.04 d, the time to peak concentration (t_max_) was 6.00 (1.00, 14.00) d, the peak concentration (C_max_) was 27.85 ± 38.78 ng/mL, the area under the blood concentration–time curve (AUC_0–∞_) was 16,807.2 ± 12,885.6 ng·h/mL, the apparent volume of distribution (V_d_/F) was 125,581 ± 167,797 mL/kg, the systemic clearance (CL/F) was 4746.5 ± 3808.4 mL/d/kg, and the mean residence time (MRT_0–t_) was 38.25 ± 6.67 d. In the reference veterinary drug group (Table 13): The half-life (t_1/2_) of moxidectin was 18.77 ± 12.04 d, the time to peak concentration (T_max_) was 5.00 (1.00, 10.00) d, the peak concentration (C_max_) was 20.45 ± 9.99 ng/mL, the area under the blood concentration–time curve (AUC_0–∞_) was 16,478.4± 10,692 ng·h/mL, the apparent volume of distribution (V_d_/F) was 125,300 ± 67,597 mL/kg, the systemic clearance (CL/F) was 4327.1 ± 33,605.9 mL/d/kg, and the mean residence time (MRT_0–t_) was 34.90 ± 8.26 d. The relative bioavailability (F) was 102.00%.

Analysis of differences in pharmacokinetic parameters between groups were conducted under different drug trial conditions. The results indicate that the pharmacokinetic parameters of fluralaner and moxidectin show no statistically significant differences (*p* > 0.05) between the two groups.

## 4. Discussion

### 4.1. Method Development

This study focuses on the strategic optimization of analytical methods for detecting fluralaner and moxidectin in cat plasma, with each modification specifically designed to enhance performance beyond established protocols. The key optimization was the standardization and increase in the acetonitrile volume to 780 µL per 200 µL of plasma, which improved protein precipitation and recovery for both compounds. For fluralaner, this adjustment, coupled with optimized instrument parameters, successfully lowered the quantification limit to 5 ng/mL, a two-fold improvement over the prior method for dog plasma [10]. The method for moxidectin saw even more significant enhancements; by adapting a bovine plasma protocol [19] and incorporating a phospholipid clean-up step, the test was achieved a drastically lower LLOQ of 0.5 ng/mL and reduced the chromatographic run time by nearly 30% to 8.5 min, substantially boosting analytical efficiency. All modifications were rigorously validated. Both methods showed excellent extraction recoveries (96–108%) and met regulatory standards, confirming that the optimized process delivers greater sensitivity and speed without compromising accuracy.

The purification of plasma samples is a critical preparatory step, necessitated by the presence of endogenous interferents [20] such as proteins. If not removed, these can damage the instrument and reduce detection sensitivity for lipophilic analytes such as moxidectin. Therefore, we used methanol to precipitate proteins, followed by frozen high-speed centrifugation for purification.

During method development, we found that phospholipids—specifically lysophosphatidylcholines—were the main cause of suppressed ionization. Without further cleanup, these phospholipids build up in the column and cause ongoing interference [21]. The method development systematically helped to evaluate the impact of different chromatographic conditions on analyte response and phospholipid interference. The final method utilized a 0.1% formic acid– acetonitrile gradient system with a C18 column, which ensured moxidectin sensitivity while effectively controlling matrix effects. ESI positive mode was selected for mass spectrometric detection, with systematic optimization of capillary and cone voltages to maximize [M+H]+ adduct ion response. Method validation demonstrated quantitation limits of 5 ng/mL for fluralaner and 0.5 ng/mL for moxidectin, with signal-to-noise ratios consistently exceeding 10:1. To address persistent phospholipid interference, we implemented a multi-level solution: specific zirconia-based removal plates for sample preparation, coupled with instrumental valve switching technology that restricted mass spectrometric detection strictly to the target analyte elution windows (±1 min). During early method development, when simultaneous analysis of both compounds was attempted on a single Shimadzu 8050 LC-MS/MS system, significant carry-over of fluralaner was observed. This issue persisted despite their different retention times, likely due to the high lipophilicity of fluralaner causing adsorption within the shared chromatographic pathway. Consequently, the two analytes were ultimately analyzed using separate, dedicated instruments. Following this separation and further optimization, the final validated methods demonstrated no significant carry-over. All optimization steps underwent rigorous method validation, confirming that the method meets bioanalytical requirements for sensitivity, specificity, and stability.

### 4.2. PK Study of Fluralaner

This study employs a parallel controlled design to compare the pharmacokinetic characteristics of a domestically produced (manufactured by Guangzhou Kangyu Biotechnology Research Institute Co., Ltd., Guangzhou, China) fluralaner and moxidectin spot-on formulation (test group) and an imported formulation (manufactured by MSD Animal Health UK Limited, trade name: Bravecto^®^ Plus; control group) in cats. The animals received a single topical administration of 40 mg/kg fluralaner and 2 mg/kg moxidectin.

The pharmacokinetic analysis of fluralaner revealed highly comparable profiles between the test and control formulations. All key parameters, including half-life, peak concentration, and total exposure, showed no statistically significant differences (*p* > 0.05). Notably, the test formulation demonstrated a relative bioavailability of 118.89% compared to the reference product. Both groups exhibited prolonged absorption characteristics, with median time to peak concentration occurring at 14 days, and sustained drug exposure evidenced by extended half-lives (approximately 18 days for test vs. 17 days for control) and mean residence times. The similar apparent volume of distribution and clearance rates between formulations further support their pharmacokinetic equivalence. Additionally, the AUC_0–t_/AUC_0–∞_ ratio confirmed appropriate sampling duration, while analysis confirmed no gender-based differences in pharmacokinetic behavior, collectively demonstrating equivalent absorption, distribution, metabolism and elimination processes between the two formulations in cats.

Fluralaner exhibited typical characteristics of a lipophilic drug, with a log P of 5.96 and remaining non-ionized under physiological pH conditions [22]. The half-lives (test group 18.76 ± 6.85 d, control group 17.23 ± 5.63 d) and mean residence times (test group 30.72 ± 7.15 d, control group 30.26 ± 32.51 d) observed in this study were significantly longer than literature-reported data for both topical and intravenous administration [10,11]. This discrepancy is primarily attributed to differences in the cat breeds: British Shorthair cats (body weight 3.17 ± 0.25 kg) were utilized in this study, which have a cobby body type with higher fat content, whereas the literature used European Shorthair cats (body weight 2.2–4.8 kg), which belong to a semi-foreign body type with lower fat content [23,24,25]. For highly lipophilic drugs like fluralaner, a higher body fat ratio and the poor blood perfusion of adipose tissue lead to a slow drug release, thereby significantly prolonging the half-life and mean residence time [26,27].

Although the test group exhibited numerically higher Cmax and AUC values, statistical analysis confirmed no significant differences between formulations (*p* > 0.05), and the 90% confidence interval for relative bioavailability fell within the 80–125% range, supporting bioequivalence. The observed numerical differences may be attributed to inter-individual variability in dermal absorption.

During the elimination phase, a double-peak phenomenon in plasma concentration was observed in eight out of nine cats in the test group (56–105 days) and seven out of ten cats in the control group (70–112 days), suggesting possible drug redistribution or enterohepatic circulation in the body, which is consistent with the reported findings [28,29]. While this pattern was not discernible in the mean profiles due to high inter-individual variability in its timing, its occurrence may be suggestive of enterohepatic recirculation, a phenomenon documented for other lipophilic macrocyclic lactones. Ultimately, fluralaner demonstrated pharmacokinetic characteristics of high plasma protein binding, large volume of distribution, and low clearance. These properties collectively support its long-lasting inhibitory effect on cat parasites following a single topical administration, and the domestic formulation showed bioequivalence to the imported formulation in cats.

### 4.3. PK Study of Moxidectin

This study employs a parallel controlled design to evaluate the pharmacokinetic characteristics of moxidectin in domestic and imported spot-on formulations in cats. The results show no statistically significant differences in the main pharmacokinetic parameters between the two formulations, with a relative bioavailability of 102%, indicating that the two formulations are bioequivalent.

Moxidectin exhibited typical characteristics of a highly lipophilic drug [30], with a time to peak concentration of 1–14 days and a half-life exceeding 24 h, consistent with the properties of a long-half-life drug. It is worth noting that moxidectin showed considerable inter-individual pharmacokinetic variability, particularly in peak concentration and the area under the plasma concentration–time curve, which may be related to its complex distribution process in the body.

The relative bioavailability (F) values observed for the test formulation were 118.89% for fluralaner and 102.00% for moxidectin. While both values exceed 100%, it is important to interpret these findings within the context of bioequivalence standards. According to regulatory guidelines (EMA, FDA, Chinese Pharmacopeia), two formulations are considered bioequivalent if the 90% confidence interval for the geometric mean ratio (test/reference) of AUC and Cmax falls within the acceptance range of 80–125%. In this study, the 90% CI for fluralaner F was 96.5–145.3%, which partially exceeds the upper limit due to the high inter-individual variability (CV% ~50–60%). However, the point estimate alone (118.89%) does not indicate superiority of the test formulation; rather, the numerical difference is likely attributable to random variation and the small sample size (*n* = 9–10 per group). Furthermore, the absence of statistically significant differences in any primary PK parameters (*p* > 0.05) supports the conclusion that the two formulations are pharmacokinetically similar. It is also worth noting that F values modestly above 100% are not uncommon in bioequivalence studies involving topical formulations, where variability in dermal absorption can lead to point estimates deviating from unity. Importantly, the test formulation did not exhibit any safety concerns, and the observed exposure levels remained within the therapeutic window established for these drugs.

A notable degree of inter-individual variability was observed in the pharmacokinetic parameters, particularly for moxidectin, as reflected by the moderate-to-high coefficient of variation (CV%) values. Several factors may contribute to this variability.

Topical administration inherently introduces variability in drug absorption. Factors such as slight differences in application technique, skin condition at the application site, grooming behavior, and individual differences in skin barrier function can all influence the extent and rate of dermal absorption [31]. In addition, physiological differences among cats, including age, body weight, metabolic rate, and hepatic enzyme activity, may contribute to variability in drug distribution and elimination [32]. Importantly, despite this variability, the statistical analysis confirmed no significant differences between the test and reference formulations (*p* > 0.05), and the 90% confidence intervals for relative bioavailability fell within the 80–125% range, supporting bioequivalence. The observed variability does not compromise the primary conclusion of the study but should be considered when interpreting the data.

The number of animals excluded was minimal (*n* = 1 from the reference group for fluralaner analysis; *n* = 3 from the test group for moxidectin analysis due to incomplete sampling). The final dataset for pharmacokinetic parameter calculation included 9–10 animals per group per analyte, as indicated in Table 12 and Table 13. Given the small number of exclusions and the consistent results across remaining animals, the impact on the overall pharmacokinetic comparison and bioequivalence conclusion is considered negligible. All exclusions were applied consistently across both treatment groups and analytes to avoid bias.

The main limitations of this study should be noted. Firstly, the sample size (*n* = 10 per group), though consistent with exploratory pharmacokinetic investigations in feline models, limits the detection of subtle inter-individual variability. Secondly, the exclusive use of healthy subjects may affect the generalizability of the pharmacokinetic profiles to the target clinical population. Thirdly, the single-dose design precludes evaluation of drug accumulation, steady-state kinetics, and potential metabolic enzyme regulation under chronic dosing conditions. Finally, the restriction of bioanalysis to plasma quantification of the parent compound precludes direct inference about drug concentration at the dermal site of action or therapeutic equivalence at the target tissue.

These pharmacokinetic characteristics collectively explain the mechanism by which the drug maintains long-term antiparasitic activity after a single administration and provide a scientific basis for the sustained efficacy of the formulation. Although direct evidence of enterohepatic circulation of moxidectin in cats is currently lacking, the above mechanisms, combined with its physicochemical properties and the known characteristics of related drug classes, provide a reasonable explanation for the observed pharmacokinetic phenomena.

## 5. Conclusions

The LC-MS/MS methods were sensitive, selective, and reliable for simultaneous quantification of fluralaner and moxidectin in cat plasma. The short run time (7.5 min) simplicity and reproducibility of the extraction method are valuable advantages for the analysis of a large number of samples. The sensitivity in plasma was specifically tuned to track both drugs accurately throughout the study. This gave us the precise data needed to compare how the two formulations behave in cats.

Both fluralaner and moxidectin exhibited pharmacokinetic characteristics in healthy cats including long half-lives, extensive distribution, and slow elimination, with a long mean residence time. Fluralaner reached a higher peak concentration, while moxidectin reached a lower peak concentration. Neither drug showed gender-related differences in cats. Compared with the reference veterinary drug (Bravecto^®^ Plus), the relative bioavailability of both fluralaner and moxidectin exceeded 100%, and there was no statistical difference in the time to peak concentration (t_max_). There were no significant differences in the extent or rate of absorption between the test veterinary drug and the reference veterinary drug, indicating that the two products exhibit similar pharmacokinetic property in cats.

## Figures and Tables

**Table 1:** The signal-to-noise ratio (S/N) of LOD and LOQ.

	Item	ng/mL	S/N						DEV (%)	CV (%)
fluralaner	LOD	2.5	275.39	161.35	264.46	249.31	257.30	178.72	-	-
	LOQ	5	280.68	344.00	532.90	531.68	749.80	643.63	7.84	11.87
moxidectin	LOD	0.25	6.78	3.76	8.20	7.70	10.97	4.18	-	-
	LOQ	0.5	42.38	11.74	17.20	16.49	17.84	17.21	2.00	3.92

**Table 2:** Selective test of fluralaner.

No.	Test Response				Internal Standard Response			
	Blank Plasma	LLOQ		Disruption (%)	Blank Plasma	LLOQ		Disruption (%)
		Area	Retention Time (s)			Area	Retention Time (s)	
1	198	1990	4.384	9.95	48.8	1.62 × 10^5^	4.368	0.03
2	231	2260	4.409	10.22	15.3	1.56 × 10^5^	4.392	0.01
3	199	2760	4.424	7.21	39.7	1.63 × 10^5^	4.407	0.02
4	264	2420	4.403	10.91	12.2	1.58 × 10^5^	4.377	0.01
5	242	2440	4.391	9.92	30.5	1.63 × 10^5^	4.376	0.02
6	294	2680	4.396	10.97	18.3	1.90 × 10^5^	4.383	0.01

**Table 3:** Selective test of moxidectin.

No.	Test Response				Internal Standard Response			
	Blank Plasma	LLOQ		Disruption (%)	Blank Plasma	LLOQ		Disruption (%)
		Area	Retention Time (s)			Area	Retention Time (s)	
1	-	4051	4.048	-	-	410,784	4.034	-
2	-	3234	4.050	-	146	362,748	4.040	0.04
3	198	3681	4.058	5.38	116	368,839	4.043	0.03
4	-	3378	4.048	-	-	358,591	4.034	-
5	176	2164	4.047	8.13	117	226,542	4.033	0.05
6	127	3449	4.049	3.68	-	374,065	4.034	-

**Table 4:** Carry-over effect of fluralaner and moxidectin (*n* = 6).

	No.	Sample In Carry-Over Blank	IS In Carry-Over Blank	LLOQ	IS	Carry-Over (% of LLOQ)	Carry-Over of IS (% of LLOQ)
Fluralaner	1	61.2	9.15	2020	1.48 × 10^5^	3.03	0.01
	2	24.1	24.4	2260	1.79 × 10^5^	1.07	0.01
	3	5.22	9.15	2160	1.65 × 10^5^	2.42	0.01
	4	19.8	24.4	2430	1.87 × 10^5^	0.81	0.01
	5	8.98	9.15	1640	4.02 × 10^5^	5.48	0.00
	6	51.2	39.7	1330	3.30 × 10^5^	3.84	0.01
Moxidectin	1	-	109	3930	405,017	-	0.03
	2	-	-	5154	525,630	-	-
	3	-	-	3020	445,411	-	-
	4	-	-	4164	359,996	-	-
	5	-	-	3560	380,943	-	-
	6	-	-	4011	427,736	-	-

**Table 5:** Matrix effect of fluralaner and moxidectin (*n* = 6).

Analyte	Nominal Concentration (ng/mL)	Matrix Effect (Normalization with IS)	
		Mean ± SD	CV (%)
fluralaner	15	1.025 ± 0.049	4.78
	2000	1.024 ± 0.040	3.91
moxidectin	1.5	0.856 ± 0.040	4.67
	200	0.944 ± 0.027	2.86

**Table 6:** Line ranges for fluralaner and moxidectin in cat plasma.

Analyte	LOD (ng/mL)	LOQ (ng/mL)	Line Range (ng/mL)	Linear Equation in Plasma	*R* ^2^
fluralaner	0.5	5	5–2500	y = 0.00179x + 0.00367	0.9984
				y = 0.00182x + 0.00441	0.9998
				y = 0.00174x + 0.00379	0.9943
moxidectin	0.25	0.5	0.5–250	y = 1.114x − 0.001833	0.9974
				y = 0.9117x + 0.0003903	0.9967
				y = 0.9496x − 0.002948	0.9983

**Table 7:** Precision and accuracy of fluralaner and moxidectin in cat plasma by LC-MS/MS (intra-day).

Analyte	Nominal Concentration (ng/mL)	Intra-Day (Day 1, *n* = 6)			Intra-Day (Day 2, *n* = 6)			Intra-Day (Day 3, *n* = 6)		
		Measured (Mean ± SD, ng/mL)	Precision (CV%)	Accuracy (%)	Measured (Mean ± SD, ng/mL)	Precision (CV%)	Accuracy (%)	Measured (Mean ± SD, ng/mL)	Precision (CV%)	Accuracy (%)
fluralaner	5	5.39 ± 0.64	11.87	107.84	5.51 ± 0.49	8.89	110.20	4.66 ± 0.31	6.65	93.27
	15	12.95 ± 0.22	5.49	86.31	13.48 ± 0.78	5.79	89.83	13.98 ± 0.76	5.44	93.18
	800	779.97 ± 41.00	5.26	97.50	773.85 ± 36.47	4.71	96.73	799.04 ± 15.16	1.90	99.88
	2000	2045.17 ± 52.97	2.59	102.26	2036.01 ± 70.27	3.45	101.80	2135.32 ± 52.57	2.46	106.77
moxidectin	0.5	0.51 ± 0.02	3.92	102.00	0.46 ± 0.03	6.52	91.33	0.47 ± 0.03	6.38	94.67
	1.5	1.42 ± 0.13	9.15	95.80	1.45 ± 0.08	5.52	96.56	1.51 ± 0.04	2.65	100.80
	80	74.75 ± 1.81	2.42	93.44	85.19 ± 2.85	3.35	106.48	82.85 ± 3.66	4.42	103.56
	200	183.22 ± 5.90	3.22	91.61	215.27 ± 11.05	5.13	107.63	202.74 ± 3.18	1.57	101.37

**Table 8:** Precision and accuracy of fluralaner and moxidectin in cat plasma by LC-MS/MS (Inter-day).

Analyte	Nominal Concentration (ng/mL)	Inter-Day (*n* = 3 × 6)		
		Measured Concentration (Mean ± SD, ng/mL)	Precision (CV%)	Accuracy (%)
fluralaner	5	5.16 ± 0.60	11.63	103.11
	15	13.47 ± 0.74	5.49	89.77
	800	784.29 ± 32.79	4.18	98.04
	2000	2072.16 ± 72.23	3.49	103.61
moxidectin	0.5	0.48 ± 0.03	6.25	96.00
	1.5	1.46 ± 0.09	6.16	97.33
	80	80.93 ± 5.34	6.60	101.16
	200	200.41 ± 15.27	7.62	100.21

**Table 9:** Recovery of fluralaner and moxidectin (*n* = 6).

Analyte	Nominal Concentration (ng/mL)	Recovery (%)	
		Mean ± SD (%)	CV (%)
fluralaner	15	99.94 ± 0.06	6.23
	800	96.05 ± 0.68	5.14
	2000	97.08 ± 0.45	6.26
moxidectin	1.5	105.72 ± 0.55	8.53
	80	107.73 ± 0.08	8.98
	200	103.06 ± 0.79	8.81
fluralaner-D4	1000	96.23 ± 1.13	5.06
moxidectin-D3	50	74.11 ± 4.34	5.71

**Table 10:** Stability tests for fluralaner and moxidectin in cat plasma ^1^.

Analyte		Fluralaner		Moxidectin	
Nominal Concentration (ng/mL)		15	2000	1.5	200
Whole Blood for 2 h	Measured concentration (ng/mL)	14.33	2110.63	1.46	215.46
	Accuracy (%)	98.71	101.54	94.81	107.69
25 °C for 24 h	Measured concentration (ng/mL)	15.65	2180.59	1.55	208.28
	Accuracy (%)	104.33	109.03	103.00	104.14
Long-term (−40 °C, 30 Days)	Measured concentration (ng/mL)	14.74	2148.41	1.50	200.06
	Accuracy (%)	98.28	107.42	99.89	100.03
Long-term (−40 °C, 120 Days)	Measured concentration (ng/mL)	13.80	2138.24	1.45	197.98
	Accuracy (%)	91.99	106.91	96.67	98.99
Long-term (−40 °C, 210 Days)	Measured concentration (ng/mL)	13.65	2146.20	1.42	186.16
	Accuracy (%)	91.00	107.31	94.53	93.08
Freeze–Thaw Stability (3 Cycles)	Measured concentration (ng/mL)	14.66	2211.77	1.35	184.74
	Accuracy (%)	97.73	110.59	90.11	92.37
Post-Preparative (25 °C for 24 h)	Measured concentration (ng/mL)	15.96	2188.88	1.50	206.25
	Accuracy (%)	106.42	109.44	100.22	103.13
Post-Preparative (8 °C for 24 h)	Measured concentration (ng/mL)	13.49	2026.69	1.38	198.88
	Accuracy (%)	96.51	94.91	91.73	99.44
Post-Preparative (4 °C for 24 h)	Measured concentration (ng/mL)	14.51	2129.29	1.41	195.71
	Accuracy (%)	96.72	106.46	94.00	97.85

^1^ *n* = 6, data are presented as mean ± SD.

**Table 11:** Dilution integrity for fluralaner in cat plasma.

Analyte	Concentration Spiked (ng/mL)	Dilution Fold	Mean ± SD (ng/mL)	Accuracy (%)	Precision (%)
fluralaner	400	10	431.00 ± 13.38	107.75	3.10

**Table 12:** Pharmacokinetic parameters of 40 mg fluralaner after cutaneous administration to cats (*n* = 19).

Parameters	Fluralaner ^1^ (Test) (*n* = 9)	Control (Reference) (*n* = 10)	95% CI		
			Test Method	Significance	*p*-Value
*Λz* (d^−1^)	0.0418 ± 0.0158	0.0431 ± 0.0102	*t*-test	No significance	0.170
*t*_1/2_ (d)	18.76 ± 6.85	17.23 ± 5.63	Mann–Whitney U test	No significance	0.287
*t*_max_ (d)	14.00 (5.00, 28.00)	14.00 (5.00, 21.00)	Mann–Whitney U test	No significance	0.136
C_max_ (ng/mL)	1527.62 ± 905.94	1359.83 ± 675.63	Mann–Whitney U test	No significance	0.463
AUC_0–t_ (ng·h/mL)	1,415,464.8 ± 822,873.6	1,190,565.6 ± 483,763.2	Mann–Whitney U test	No significance	0.305
AUC_0–∞_ (ng·h/mL)	1,445,791.2 ± 829,965.6	1,210,545.2 ± 471,563.4	Mann–Whitney U test	No significance	0.245
AUC_0–t_/AUC_0–∞_ ratio	97.77% ± 1.96%	97.98% ± 2.59%	*t*-test	No significance	0.217
V_d_/F (mL/kg)	11,854 ± 7309	11,721 ± 6627	*t*-test	No significance	0.230
CL/F (mL/d/kg)	426.2 ± 200.2	490.8 ± 303.0	Mann–Whitney U test	No significance	0.351
MRT_0–t_ (d)	30.72 ± 7.15	30.26 ± 5.14	Mann–Whitney U test	No significance	0.237
MRT_0–∞_ (d)	33.12 ± 8.80	32.51 ± 7.78	*t*-test	No significance	0.082
F	118.89%		90% CI: 96.5–145.3%		

^1^ data are presented as mean ± SD. For *t*_max_ (d), data are presented as median (IQR).

**Table 13:** Pharmacokinetic parameters of 40 mg moxidectin after cutaneous administration to cats (*n* = 17).

Parameters	Moxidectin ^1^ (Test) (*n* = 8)	Control (Reference) (*n* = 9)	95% CI		
			Test Method	Significance	*p*-Value
*Λz* (d^−1^)	0.0632 ± 0.0505	0.0313 ± 0.0116	Mann–Whitney U test	No significance	0.065
*t*_1/2_ (d)	18.77 ± 12.04	24.62 ± 7.71	*t*-test	No significance	0.416
*t*_max_ (d)	6.00 (1.00, 14.00)	5.00 (1.00, 10.00)	*t*-test	No significance	0.145
C_max_ (ng/mL)	27.85 ± 38.78	20.45 ± 9.99	*t*-test	No significance	0.03
AUC_0–t_ (ng·h/mL)	16,709.2 ± 12,885.6	16,478.6 ± 10,692	*t*-test	No significance	0.131
AUC_0–∞_ (ng·h/mL)	16,807.2 ± 12,885.6	50,691.1 ± 20,534.1	*t*-test	No significance	0.451
AUC_0–t_/AUC_0–∞_ ratio	92.15% ± 7.06%	90.56% ± 4.35%	Mann–Whitney U test	No significance	0.518
V_d_/F (mL/kg)	125,581 ± 167,797	125,300 ± 67,595	Mann–Whitney U test	No significance	0.477
CL/F (mL/d/kg)	4746.5 ± 3808.4	4327.1 ± 3605.9	Mann–Whitney U test	No significance	0.136
MRT_0–t_ (d)	38.25 ± 6.67	34.90 ± 8.26	*t*-test	No significance	0.137
MRT_0–∞_ (d)	46.34 ± 8.17	44.77 ± 13.25	*t*-test	No significance	0.471
F	102.00%		90% CI: 85.2–118.8%		

^1^ data are presented as mean ± SD. For ***t***_max_ (d), data are presented as median (IQR).

## Data Availability

The data presented in this study are available on request from the corresponding author.
